# Projecting temperature-related dengue burden in the Philippines under various socioeconomic pathway scenarios

**DOI:** 10.3389/fpubh.2024.1420457

**Published:** 2024-12-23

**Authors:** Xerxes Seposo, Sary Valenzuela, Geminn Louis C. Apostol, Keith Alexius Wangkay, Percival Ethan Lao, Anna Beatrice Enriquez

**Affiliations:** ^1^Ateneo School of Medicine and Public Health, Pasig, Metro Manila, Philippines; ^2^Department of Hygiene, Graduate School of Medicine, Hokkaido University, Sapporo, Hokkaido, Japan; ^3^Department of Global Human Development, School of Foreign Service, Georgetown University, Washington, DC, United States

**Keywords:** Philippines, dengue, temperature, economic burden, projections, climate change

## Abstract

**Introduction:**

As climate change advances, the looming threat of dengue fever, intricately tied to rising temperatures, intensifies, posing a substantial and enduring public health challenge in the Philippines. This study aims to investigate the historical and projected excess dengue disease burden attributable to temperature to help inform climate change policies, and guide resource allocation for strategic climate change and dengue disease interventions.

**Methods:**

The study utilized established temperature-dengue risk functions to estimate the historical dengue burden attributable to increased temperatures. Future projections were derived using Coupled Model Intercomparison Project Phase 6 (CMIP6) Shared Socioeconomic Pathway (SSP) scenarios to estimate the excess dengue burden on a national scale. Current health burden estimates were calculated by charting the attributable fraction per epidemiological week against the exponential risk function.

**Results:**

Projections indicate a substantial increase in temperature-related dengue incidence across all SSP climate scenarios by 2100. Between 2010-2019, 72.1% of reported dengue cases in the Philippines were attributable to temperature, demonstrating that temperature is a significant driver in dengue transmission. The highest attributable fractions were observed between the warm-dry season to early rainy season (Epi Weeks 15–25). Southern, periequatorial areas, particularly those undergoing rapid urbanization, had the highest temperature-related dengue incidence.

**Discussion:**

The findings emphasize the critical interplay between climate change and socioeconomic factors in shaping future dengue risk. By incorporating future climate scenarios and provincial-level projections, this study provides valuable insights for policy planning, early warning systems, and public health programming. Strengthening health infrastructure, promoting sustainable urban development, and implementing effective vector control measures are crucial to mitigating the future dengue burden in the Philippines.

## 1 Introduction

Dengue fever continues to be a global threat worldwide, with the World Health Organization (WHO) estimating about half of the world’s population being at risk, and an estimated 100–400 million infections occurring each year (1). Dengue cases tend to be under-reported, as a vast majority of these cases are asymptomatic, approached with variable treatment-seeking behavior, or misdiagnosed as other febrile illnesses (1–3). Dengue fever can be fatal, but even when it is not, individuals and communities face a substantial risk of financial burden when afflicted by the disease, especially in tropical and sub-tropical climates where healthcare delivery tends to be underdeveloped.

Dengue is especially a concern in South East Asia, where as much as 1.3 billion of the 3.5 billion people living in dengue endemic countries are located (4).

The Philippines is no exception, where dengue is endemic, with 220,705 cases reported in 2022 (5), and a 94% increase in incidence during the 1st quarter of 2023 compared to the same period of the previous year (6). All four serotypes of dengue exist in the Philippines, however, DENV-1 2, and 3 have predominated since 1995, with sporadic instances of DENV-4 (7). Primary infection offers immunogenetic protection against homologous serotypes, but increases severity upon secondary heterogenous serotypes especially among children (8). As a notifiable disease, dengue is part of the surveillance program of the Philippine Integrated Disease Surveillance and Response (PIDSR) manual. The WHO criteria is used to classify patients as suspected (acute febrile illness coupled or with or without warning signs of dengue), probable (suspected symptoms with supporting laboratory tests, i.e., complete blood count, positive NS1 antigen test, or positive dengue antibody tests) or confirmed dengue (suspected symptoms with positive viral culture or PCR results). Severe dengue cases include those with severe plasma leakage, hemorrhage, or organ impairment (9). Probable, confirmed, and severe cases of dengue are then collated and reported by the Philippines Department of Health Epidemiological Bureau (7, 8). Humans serve as the main reservoir host in dengue endemicity, but recent studies have found possible enzootic transmission (10). Currently, there are limited studies that investigate the role of non-human reservoirs in dengue transmission and disease burden in the Philippines.

The looming threat of climate change poses the growing concern that this could affect the already high rates of dengue transmission in the country. The primary vector of dengue is *Aedes aegypti* and *Ae. albopticus* (10–12). In a systematic review, Naish et al. (11) noted that dengue is highly sensitive to climatic conditions, particularly with temperature, rainfall, and relative humidity, albeit the relationship being characterized by non-linear dynamics due to the vector’s biological needs and life-cycle. The influence of temperature on vector fecundity and viability have been well-demonstrated as a parabolic curve. Within certain ranges, increased temperatures have been seen to decrease vector incubation time and increase mosquito population. Beyond these temperatures, vector viability decreases (11–14). On the other hand, Fan et al. (15) reported that dengue transmissions increase steeply as the temperature increases between 22 and 29°C. The study purports that this trend may pose a greater concern for regions in sub-tropical and temperate climates, rather than in tropical climates. However, a review by Li et al. (16) showed that increasing temperature increases risk of dengue even in tropical countries. Hence, as temperatures are likely to rise from climate change, there is a growing need for robust dengue projections to anticipate and mitigate the potential future temperature-driven dengue burden. Accurate projections can guide policy makers and healthcare professionals to implement appropriate interventions and efficiently allocate resources to combat dengue.

There have been several studies that have attempted to project potential dengue-burden. Xu et al. (17) noted 16 studies that have projected dengue burden by the year 2050. The same review found Representative Concentration Pathway (RCP) 4.5, 6.0, and 8.5 as the most common climate change scenarios employed. Outcome measures were also diverse, as some studies projected the future number of dengue cases, while others projected spatial distribution, population at risk of dengue, or future epidemic risk potential (probabilities). Another review by Soneja et al. (18) identified 35 studies with similar findings, generally suggesting increased risk of dengue through different climate change scenarios. There were however several studies within the review that found inconsistent results or even decreased risk of dengue that was attributed to either spatial redistribution (e.g., increases in temperate areas but decrease elsewhere) or negative effects to the dengue life-cycle directly.

Multiple approaches have been employed to project and assess the risk of dengue in the future. These methods included probabilistic outcome based on dengue simulations (DENSiM) (19), l human-to-mosquito and mosquito-to-human infection probabilities (20), and machine learning in conjunction with entropy-based modeling (21), among others. The most common projection, however, was done through estimation of the number of dengue cases (22–24). Additionally, many previous studies estimated the current and future temperature-dengue burden as an average effect by using the pooled or non-effect modified association (17, 18, 22–24). While the average effect can give an idea about the general dengue health burden, one would expect that as a result of temperature, in several instances, this health burden may not necessarily reflect that of areas with unique socio-economic features. Large disparities in population distribution as well as urbanization, alongside healthcare spending have been noted to alter the temperature-dengue association (3, 13).

In light of the upward trajectory of climate change, strategic resource allocation becomes imperative to combat the escalating threat of dengue fever, in countries that suffer high dengue disease morbidity and mortality such as the Philippines. Projecting dengue incidences assumes critical significance not only for risk assessment but also for policy formulation, thereby facilitating the development of robust contingency plans, surveillance frameworks for epidemic preparedness, algorithmic alert systems, vector control strategies, and effective clinical management protocols for substantial caseloads (25–27). This study aims to provide data-driven guidance to bolster climate change mitigation as a response to the burden of infectious, vector-borne diseases, including dengue fever, which is included in key national policies and frameworks such as the National Environmental Health Action Plan 2023–2030 (28), National Climate Change Action Plan 2011–2028 (29), and Philippine Development Plan 2030 (30). While existing studies for dengue modeling in the Philippines have predominantly focused on localized or regional analyses (27, 31–34), the absence of a nationwide projection system hampers the efficacy of dengue management efforts on a broader scale. By harnessing predictable and possible climatic scenarios, a nationally-scoped projection system promises to furnish the requisite tools for enhancing dengue mitigation strategies across the archipelago, thereby facilitating a concerted nationwide endeavor in combating this pressing public health challenge.

## 2 Materials and methods

### 2.1 Data instrumentation

Dengue data was obtained from weekly notifiable dengue cases per province (*n* = 81) reported to the Philippines’ Department of Health-Epidemiological Bureau from 2010 to 2019. These only included cases reported in tertiary, secondary, or municipal health centers within the time frame, not accounting for asymptomatic dengue cases or those managed solely at home. Provinces with less than 300,000 population (*n* = 20) were excluded from the analysis due to lack of statistical power, which would subsequently over or underestimate the results otherwise. Since there is a lack of background weather monitoring stations for each of the provinces in the country, we utilized the ERA5-Land hourly data, that was subsequently aggregated by day, and finally aggregated by epidemiological week. For more details on the data agreement between ERA5-Land and locations with actual monitoring stations, please refer to the previous study (13). Hereon, epidemiological week is interchangeably utilized with week. The data used for provincial healthcare spending were yearly, province-specific, from the latest national survey data from the Philippine Statistics Authority (35).

### 2.2 Risk estimation

The temperature-dengue risk functions were generated from the previous study by the authors (13). Dengue data was assumed to follow a negative binomial distribution. Subsequently, we included a crossbasis term for temperature and adjusted for several covariates as shown in Equation 1.


(1) $$\begin{array}{l} Y_{t,i}∼\mathrm{Negative}\;\mathrm{binomial}\\ \begin{array}{l} Y_{i,t}∼\alpha +\log\mathrm{pop}+\mathrm{cbtemp}+s\left(\mathrm{Epiweek},k=4\right)\\ \;+\left(1|\mathrm{Year}\right)+\left(1|\mathrm{Pr}\mathrm{ovince}\right)+Y_{t-1,i}+\varepsilon \end{array} \end{array}$$

Yt,i is the weekly dengue incidence in time (t) of province (i). The weekly dengue incidence is assumed to follow a negative binomial distribution, which is similar to previous studies (36). *α* is the intercept. *Logpop* is the log of the population, which is an offset of the changing population sizes (16). *cbtemp* is a crossbasis function of the exposure and lag dimensions of temperature, with both dimensions parameterized with 4 degrees of freedom (df) using natural cubic splines, respectively. The maximum lag was set at 18 weeks (or 4.5 months), with an adjustment of the seasonality of dengue cases through a 4 df. Owing to the variations in the annual distribution of cases per year (Supplementary Figure S2), we added a random effect term of year; *(1|Year)*. A random effect term of Province *(1|Province)* was also added to emulate a (partial) pooling effect in estimating the nationally representative association. Several studies have noted the potential immunity in the population, which could inflate the susceptible population in the subsequent time scale. To model this phenomenon, Imai et al. (37) utilized the previous time scale’s case as a potential adjustment. Here, we adjusted for the previous week’s case; Y_t − 1,i_. *ε* is the error term. Sensitivity analyses were also conducted by adjusting the effect of humidity and precipitation in the model; results were found to be robust even after adjustment (13), as a result, humidity and precipitation were excluded from the risk function. For additional information about the parameterization of the model, please see the previous study (13). In the previous study, the temperature-dengue risk function was noted to be non-linear, for simplicity and ease of comparison with future temperature-related dengue burden, we re-fit the shape to an exponential risk function (as shown in Supplementary Figure S1).

### 2.3 Estimating the historical, temperature-related dengue burden

Charting against the temperature-dengue risk function in Supplementary Figure S1, we were able to estimate the temperature-dengue attributable fraction (AF) across the actual temperature range per epidemiological week. The week-specific AF was then multiplied with the week-specific dengue incident cases to generate the week-specific attributable number (AN). The week-specific AN was then aggregated per province and across the country as well as sociodemographic factors in yielding the current temperature-dengue health burden. Equation 2 was used to derive the AF and AN using the following specifications:


(2) $$RR=\exp^{\beta x}$$

$$AF=\frac{\left(RR-1\right)}{RR}$$

$$\mathrm{AN}=AF\times \mathrm{Mor}b_{\mathrm{baseline}}$$

The exponential of the multiplicative output between β , beta coefficient derived from the linearized function, and x, the weekly temperature, yields the relative risk (RR). Attributable fraction (AF) can then be estimated by utilizing the generated RR. Attributable Number (AN) is calculated by taking the multiplicative output of baseline morbidity ( $\mathrm{Mor}b_{\mathrm{baseline}}$ ) and AF. Here, baseline morbidity is the weekly number of dengue cases of the area divided by its total population.

### 2.4 Estimating the future, temperature-related dengue burden

We utilized the Coupled Model Intercomparison Project Phase 6 (CMIP6) Shared Socioeconomic Pathway (SSP) scenarios of SSP1-2.6, SSP 2–4.5, SSP3-7.0, and SSP5-8.5 in projecting the future dengue burden. The choice of these four scenarios, with the exclusion of SSP1-1.9, is consistent with the approach of past studies done on vector-borne diseases, and is similar with the contexts in the Philippines (38–41). This also took into consideration the limited practicality and improbability of this highly ambitious scenario (38, 39, 42). From the currently available CMIP6 Global Circulation Models (GCM) data (in netCDF), only 11 GCMs, namely, AWI-CM-1-1-MR, BCC-CSM2-MR, CESM2, GFDL-ESM4, INM-CM4-8, INM-CM5-0, IPSL-CM6A-LR, MIROC6, MPI-ESM1-2-LR, MRI-ESM2-0, and NorESM2-MM out of the 27 GCMs had complete observations for historical and the various SSPs. Both historical and SSP scenarios were at 1.0° (~100 km) daily resolution for near-surface air temperature. We utilized google earth engine (GEE) to efficiently post-process the netCDF files by using the computing capacity of the GEE servers. Province-level shapefiles were uploaded to GEE, which was then used as boundaries to summarize the exposures by the province polygon. We took the daily ensemble mean of the 11 GCMs for each of the SSP scenarios by province.

Population projections per province were sourced from NASA’s Socioeconomic Data and Applications Center (SEDAC), unit of the Earth Institute at Columbia University (43, 44). These datasets provide global, spatial population and urban land projections in a unit of persons per square kilometer, every year from 2000 to 2,100 based on SSPs, v1.01. Previous studies have used these population projections in modeling and projecting dengue incidence in the USA (45), China (46, 47), Mexico (48), India (49), and Vietnam (50) due to its robust, fine-scale datasets. The use of more granular, fine-scale datasets (i.e., daily or weekly cases) allow representation of short-term changes, as opposed to yearly datasets. Future socio demographic-specific excess dengue AN is calculated using the following Equation 3.


(3) $$RR_{i,j,k}=\exp^{\beta_{k}\left(x_{SSP_{i},j}-x_{\mathrm{historical},j}\right)}$$

$$\begin{array}{l} \mathrm{Excess}\;AN_{2030-2100,i,j}=\left(\frac{\left(RR_{i,}-1\right)}{RR_{i,j}}\right)\\ \times \left[\left(\frac{\mathrm{Dengue}\;\mathrm{case}s_{\mathrm{baseline}}}{\mathrm{populatio}n_{\mathrm{baseline}}}\right)\times \left(\mathrm{populatio}n_{2030-2100}\right)\right] \end{array}$$

$RR_{i,j}$ is the relative risk generated from the combination of SSP scenario *i* of province *j*, generated from multiplying β , the beta coefficient derived from the linearized function in Equation 1, and the difference in the historical and SSP-specific temperature, $x_{SSP_{i},j}-x_{\mathrm{historical},j}$ . $\mathrm{Dengue}\;\mathrm{case}s_{\mathrm{baseline}}$ as the name suggests are the dengue cases in the baseline year, in this case 2010–2019. We took the annual mean of the dengue cases across the period, essentially yielding annualized dengue incidence. This annualized dengue incidence was then divided by the 2015 population ( $\mathrm{populatio}n_{\mathrm{baseline}}$ ) to generate the dengue morbidity rate at baseline. Medium variant population estimates were then taken based on SEDAC provided decadal data between 2030 to 2,100 ( $\mathrm{populatio}n_{2030-2100}$ ). Lastly, by taking the multiplicative output of the aforementioned variables, the future socio demographic-specific excess dengue AN can then be generated. Data pre-processing was implemented using both GEE and R statistical programming. Subsequent current and future health burden estimation was carried out using R statistical programming.

The distinction between projections for different timeframes, such as up to 2,030, 2,040, and 2,100, lies in their utility for providing and addressing both medium-term, and long-term health challenges and strategically aligning with national action plans. The Philippines’ National Climate Change Action Plan (29) and Philippine Development Plan (51) will be due for review and evaluation by the year 2028 and 2040, respectively. Projections in the year 2,100 will allow us to plan and mitigate possible long-term environmental health issues according to different climate scenarios.

### 2.5 Using healthcare spending to decrease dengue incidence

In seeking to address climate change projections, the authors aimed to explore the viability of leveraging increased provincial healthcare spending as a means to reduce dengue incidence. Provincial healthcare spending was a selected socioeconomic factor from the previous study (13), included due to their strong association with dengue incidence according to relevant literature (52, 53). We excluded average household size, poverty incidence, proportion of urban spending, and population density due to the overlinearized risk functions during a brief sensitivity analysis. Limitations in estimating the risk function due to the differences in shape patterns would lead to misleading and over or underestimation of the results, hence exclusion from the analysis.

## 3 Results

### 3.1 Historical attributable fraction of temperature-related dengue cases per province (2010–2019)

**Figure 1:**
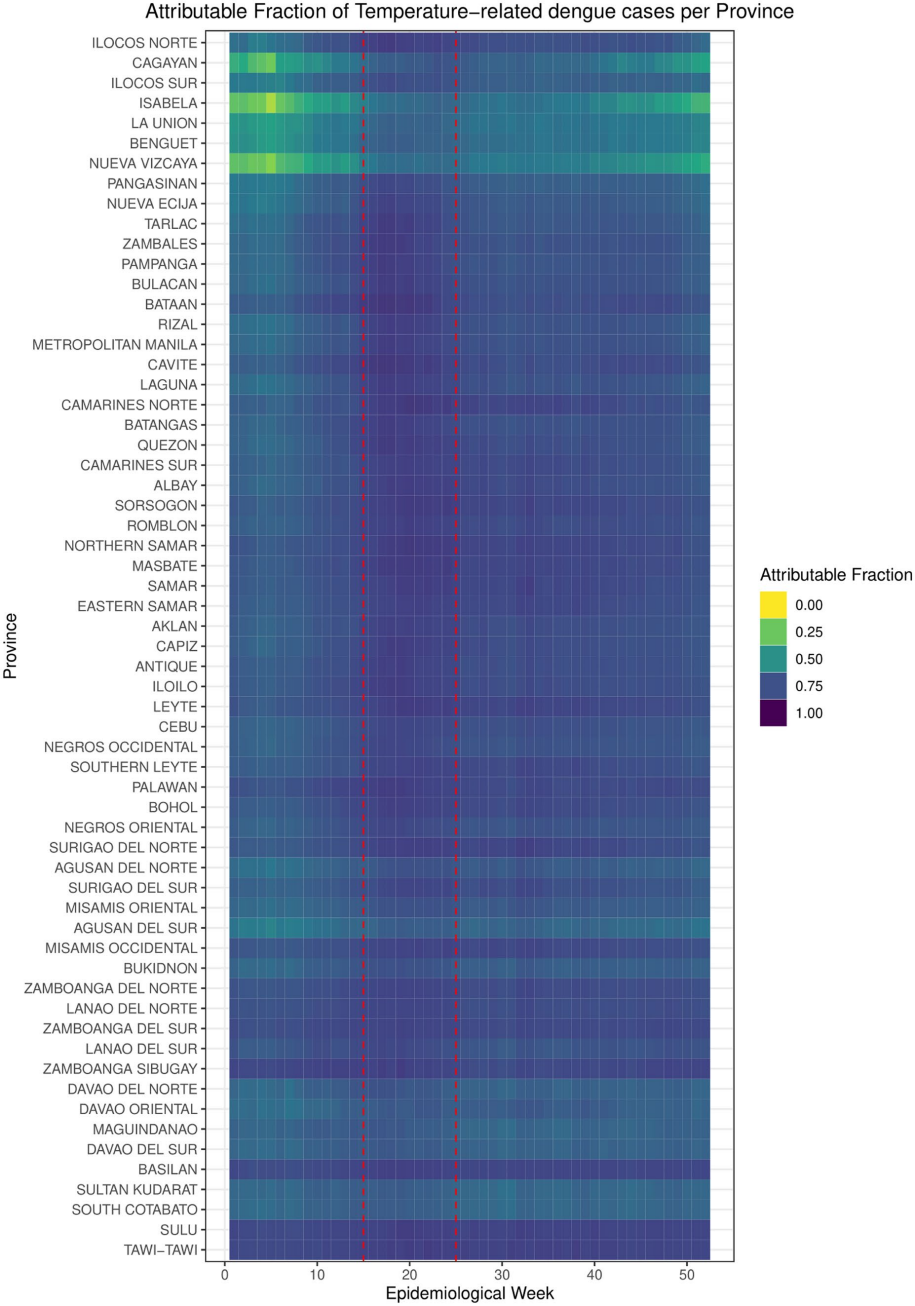
Attributable fraction of temperature-related dengue cases per province.

Based on the calculated risk functions, the attributable fraction (AF) of dengue cases attributable to increased temperature per province from 2010 to 2019 was calculated at 72.2%, with a range of 45.6–80.6% of reported cases. This is illustrated in Figure 1, with Epiweek represented by the x-axis and the provinces listed in the y-axis in increasing latitude. The degree of attribution is represented by the color gradient with yellow representing the lowest AF (0.0, 0%) and purple with the highest AF (1.0, 100%). Visually, the highest AF is highest around Epiweeks 15 to 24 (1st week of April to 2nd week of June, enclosed in the red box), which falls in the middle of warm-dry season and lasts until the beginning of the rainy season.

### 3.2 Historical sum-weekly national dengue cases and attributable number by temperature (2010–2019)

**Figure 2:**
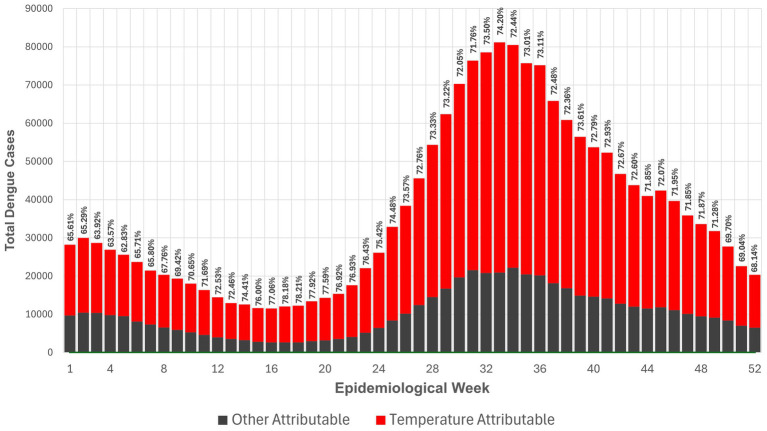
Attributable number and attributable fraction of temperature-attributable dengue cases per Epiweek across provinces from 2010–2019.

A total of 1,909,209 dengue cases were reported between January 2010 to December 2019, and 1,370,009 cases (72.1%) were attributable to temperature. Figure 2 shows the attributable number (AN) and AF of temperature-attributable dengue cases per Epiweek across all provinces within the 10-year time period. In absolute terms, the average temperature-attributable number of dengue cases was 26,346 per Epiweek (72.1%), with a range of 8,828 cases (Epiweek 5) to 60,216 cases (Epiweek 33). The AN was lowest during Epiweeks 12–20, falling within the warm-dry months of March to May, and highest during Epiweeks 30–36 (late August to end of September), which fall within the rainy season. Though the highest AN occurred during the rainy months, the percentage of temperature-attributable dengue cases (AF) saw a sharp increase during the summer months (Epiweek 14) before decreasing toward the beginning of the rainy season (Epiweek 24).

### 3.3 Annualized attributable number of incident dengue cases per 100,000 population (2010–2019)

**Figure 3:**
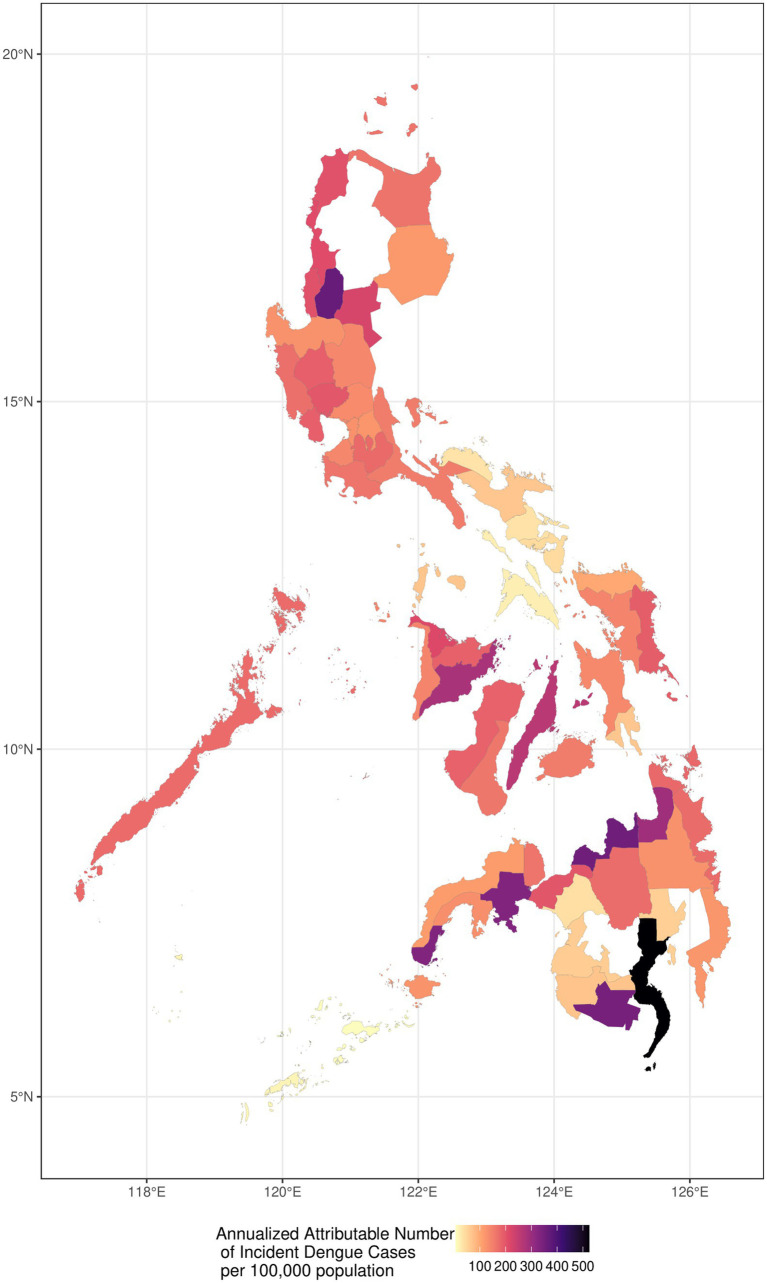
Historical annualized dengue incidence and burden by province from 2010 to 2019.

Geographically, Figure 3 depicts the historical annualized dengue incidence and burden by province from 2010 to 2019 per 100,000 population, to compare the AN per province with consideration to the provincial population. Provinces with high incidence are shown with darker gradients, whereas those lighter in color represent lower incidence. While there is no consistent geographical pattern, it is notable that southern, peri-equatorial regions generally had substantially darker patches, indicating higher temperature-related burden than that of northern locations. The provinces with the highest annualized attributable number of temperature-related dengue incidence (number of cases per 100,000 population) were Davao del Sur (505 cases), followed by Misamis Oriental (342 cases), and South Cotabato (335 cases). The provinces with the lowest incidence include Lanao del Sur (37 cases), Tawi-Tawi (16 cases), and Sulu (6 cases). Among the 61 provinces identified in our study, eight of which have a historical annualized attributable number of greater than 250 per 100,000 population of incident dengue cases, falling within the greater half of the gradient. Of these eight provinces, five of which are situated in Mindanao, namely Agusan del Norte, Davao del Sur, Misamis Oriental, South Cotabato, and Zamboanga del Sur. Two are located in the Visayas region, namely Cebu and Iloilo. Lastly, Benguet province, located in the northern part of the Philippines was the lone area with a darker gradient in the island of Luzon.

### 3.4 Future dengue projections, by SSP

**Figure 4:**
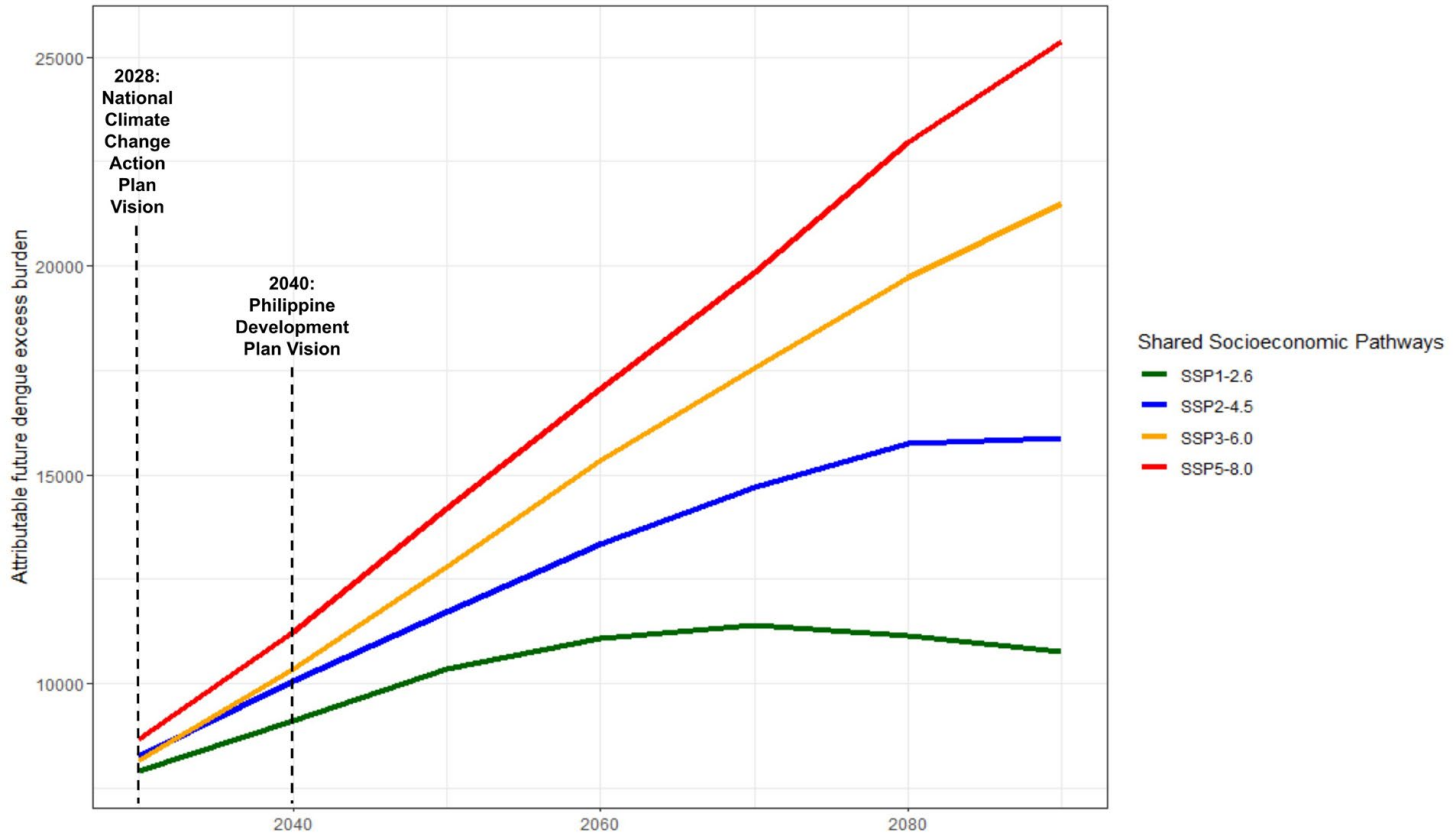
Nationally-representative projected dengue incidence projections under different Shared Socioeconomic Pathways (SSPs).

Four distinct SSP scenarios are visualized in Figure 4, ranging from relatively optimistic (SSP1-2.6, Sustainable development scenario) to least optimistic (SSP5-8.5, Fossil-fueled development scenario), reflective of diverse outcomes contingent upon the efficacy of climate mitigation measures from 2,030 to 2,100 (54). Across all SSPs, a consistent upward trend in dengue incidence is observed, persisting until approximately 2070. However, deviations emerge thereafter, contingent upon the realization of specific SSPs. Notably, SSP 1–2.6 forecasts a gradual decline in dengue cases post-2070, while SSP 2–4.5 indicates a plateauing of incidence by 2080. Conversely, SSP 3–6.0 and 5–8.0 sustained escalations in excess dengue burden beyond 2,100, with no indication of plateauing or decreasing. Dengue incidence under SSP5-8.0 exhibited +135.85% higher incidence rates than the most conservative SSP 1–2.6 by 2,100. The differences in excess dengue incidence across SSPs can be found in Supplementary Table S1. Noteworthy temporal benchmarks at 2030 and 2040 are highlighted, aligning with the forthcoming iterations of the National Climate Change Action Plan and Philippine Development Plan. These junctures offer strategic opportunities for the implementation of targeted climate interventions, pivotal in steering trajectories toward more favorable SSP outcomes, underscoring the critical intersection between national climate strategies and dengue mitigation efforts.

### 3.5 Attributable dengue incidence projections by SSPs according to healthcare spending

**Figure 5:**
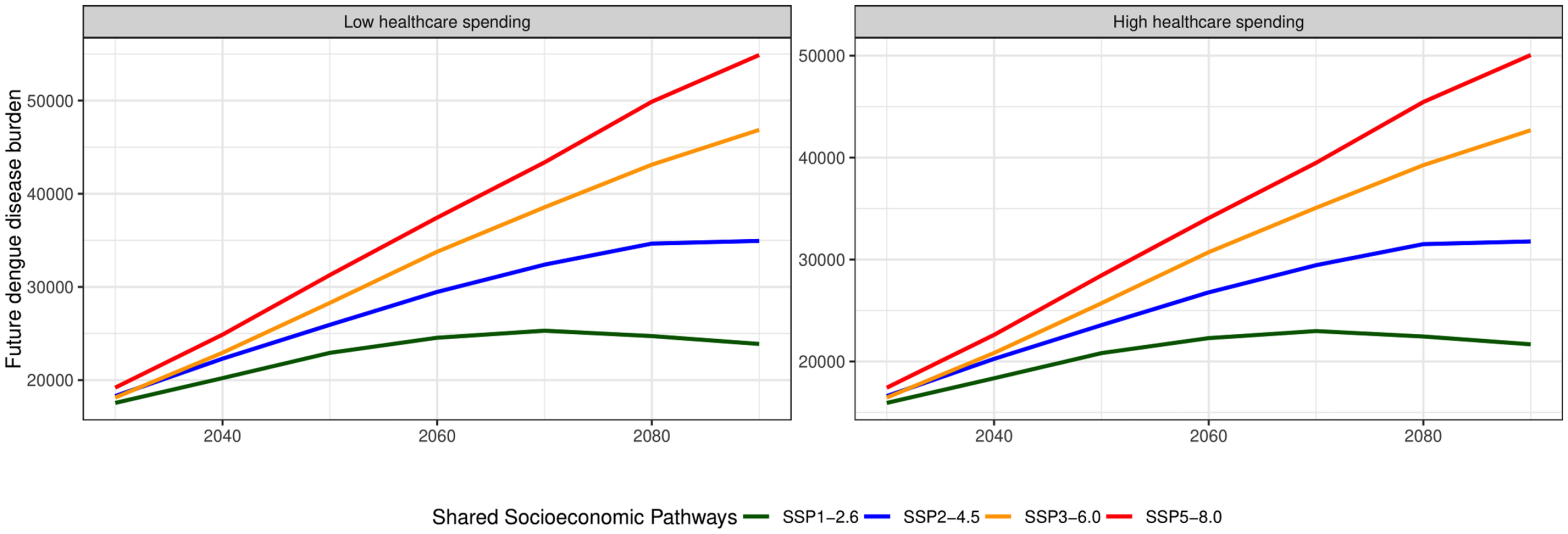
Nationally-representative attributable dengue incidence projections by SSPs according to healthcare spending.

Interestingly, minimal disparity is observed between low and high healthcare spending regions in terms of excess dengue burden trends. Figure 5 depicts the projected attributable dengue incidence under different Shared Socioeconomic Pathways (SSPs) according to high or low provincial healthcare spending. Provinces with lower healthcare spending consistently exhibited a higher incidence of dengue cases, ranging from 1,000 to 5,000 more excess temperature-attributable dengue cases compared to provinces with higher healthcare spending across all SSPs until the year 2,100.

## 4 Discussion

### 4.1 Seasonal dynamics of dengue transmission

Our findings indicated a notable seasonal trend between increased temperature and dengue incidence, particularly during the summer months from the first week of April to the second week of June (Figure 1). During this time, approximately 72.2% of dengue cases within this period were attributable to elevated temperatures. While dengue is known to be endemic, year-round in the Philippines (55, 56), the results demonstrate that the warm-dry summer season still exists as a critical period for dengue transmission, challenging the conventional notion of dengue primarily as a disease of the rainy season (31, 33). Similar results have also been observed in Argentina (57) and Vietnam (58). Household practices may contribute to dengue incidence in the summer, especially in more rural areas where the storage of extra water may lead to more mosquito breeding habitats (11, 59). Our results, though have adjusted for precipitation as a covariate, potential change in the attribution due precipitation-focused analysis may provide insightful results.

Nation-wide average attributable fraction of dengue cases due to temperature each week is at 72.2% (Figure 2). The lowest attributable fraction is seen at epidemiological week 5 (at 62.83%), while the highest is at week 18 (at 78.21%). This conveys that temperature is the largest contributor to the number of dengue cases, regardless of the time of year. This also translates to an attributable number of cases of 26,346 per week caused by temperature alone. Mechanisms offered to explain these findings show that higher temperatures can shorten the external incubation period and increase population transmission rates among *Aedes albopictus* mosquitoes (60). In addition, vector competence was found to increase with higher temperatures, as indicated by the rising titers of DENV-2 in the salivary glands of *Aedes albopictus*, and increasing rates of dengue virus replication in mosquitoes (61). Similar patterns have been observed in *Aedes aegypti* mosquitoes for the DENV-1 and DENV-2 serotypes (62). A systematic review using wing morphology as a basis also showed that the wings of *Aedes* mosquitoes tend to be shorter at higher temperatures, with smaller adults associated with faster larval development. Consequently, smaller *Aedes* females are more efficient at selecting breeding sites, contributing to a higher rate of dengue virus infection and spread (63).

A sharp increase is observed in temperature-attributed cases at Epiweek 24, peaking at Epiweek 32 before steadily declining until the end of the year. This trend corresponds to the rainy season, between the months of June to August. While the highest attributable fraction occurred in the summer months, the highest attributable number was observed in the rainy season. The peak attributable number is consistent with literature that describes dengue as having a three-month lag, resulting from incubation periods in the vector-pathogen-host transmission cycle (58, 64, 65). This potentially explains how high temperatures in the summer months of March to May can drive increases in dengue incidence early into the rainy season. Although precipitation was excluded in the analyses of this study, the increasing frequency of non-monsoon rains during the late dry season (December to February) may also contribute to spikes in dengue incidence during the warm-dry months of March to May (66).

In response to the increased temperatures during the dry months, one study found that the eggs of *Aedes aegypti* mosquitoes can undergo extended quiescence, allowing larvae to survive by relying on stored reserves. However, this prolonged dormancy depletes their nutrients, especially lipids, making newly hatched larvae more vulnerable to environmental stress. Increased temperatures during the dry season further exacerbate these effects by reducing larval fitness and survival (55, 67).

Given that *Ae. aegypti* larvae are more physiologically compromised after this extended quiescence, especially in high-temperature environments, control strategies should target these vulnerable larvae year-round (55). Such findings have implications on current public health policy and practices in the Philippines, whose current efforts at promoting dengue mitigation strategies such as the enhanced 4S strategy are deployed mainly at the onset of the rainy season. As rising global temperatures are reducing seasonal differences in mosquito populations, such a strategy would be more effective if implemented throughout the entire year. Continuous intervention could significantly curb mosquito populations and help suppress dengue transmission before the wet season begins.

### 4.2 Regional hotspots of dengue incidence

Among those classified as highly urbanized cities in the Philippines, nine are situated among the top eight provinces with the highest annualized attributable number of incident dengue cases (Figure 3) (68). The Mindanao region comprises majority of these cities, where five out of the nine cities (province) fall under this classification: Butuan City (Agusan del Norte), Davao City (Davao del Sur), Cagayan de Oro City (Misamis Oriental), General Santos City (South Cotabato) and Zamboanga City (Zamboanga del Sur). This remains consistent with our previous study showing those with the highest dengue incidence are highly urbanized provinces (13). Exploring the impact of urbanization on dengue, factors like rapid urban growth without proper infrastructure such as water systems and waste management, create breeding grounds for dengue vectors (58). In Davao del Sur, where the highly urbanized city of Davao is located, remains to be the province with the highest temperature-attributable dengue incidence. Simultaneously, a study conducted by the Japan International Cooperative Agency (JICA), identified the worsening conditions of water bodies in the city, which is quite common in its thriving urban growth centers where water utilization is high. Despite high utilization, wastewater treatment is inadequate (69). Local studies have also highlighted the urban water system’s very high vulnerability to climate change, noting concerns over aging infrastructure and the absence of effective waste management (70). Additionally, waste segregation remains to be a challenge in the Philippines, wherein dry waste such as plastic containers is often not separated from wet waste (71). Such conditions provide more places for stagnant water to, further exacerbating breeding conditions for the *Aedes* mosquito (72).

Beyond issues of water quality and sanitation, the Philippines (73), more so in the Mindanao provinces like Davao del Sur and South Cotabato, where the highest burden of temperature-attributable dengue cases are concentrated, face a water crisis. These provinces have endured water scarcity, and face a high risk for drought (74–76). In fact, the entire island of Mindanao is categorized as very high risk for climate change-induced drought, putting it as the most vulnerable among all the regions (77). These conditions force practices of water rationing and the use of storage containers for water, inadvertently creating mosquito breeding sites (78). Such practices not only impact households regardless of their water storage methods but also serve as indirect channels for dengue transmission, highlighting the importance of addressing both environmental and socioeconomic factors in mitigating dengue risks (79, 80).

### 4.3 Projecting dengue incidence

Our results in Figure 4 emphasize the projections of excess in dengue incidence in three different points in time. Extreme SSP scenarios, particularly SSP5-8.5 resulted in an excess of as much as 10% of current levels (additional 30,000 dengue cases) by 2,100. Using the middle-of-the-road (SSP2-4.5) as a reference scenario and assuming that trends in global CO2 emissions and socio-economic progress continue at current trends, an additional 16,000 cases will still be anticipated by the end of 2,100, with no clear sight of decline or return to current levels in the short-to-medium term. Whereas, the sociodemographic modified-dengue health burden varied across variables of interest, with a majority of the health burden to be magnified in the future. In a recent Southeast Asian study, Colon-Gonzalez and colleagues (81) observed that the multi-GCM multi-scenario ensemble mean dengue predictions in 2050 for several countries, including the Philippines, will increase. If the increase in emissions are maintained at current levels (SSP3-7.0), the Philippines will anticipate a peak of nearly 108,000 total dengue cases by 2080 and plateauing thereafter (61). The future burden of dengue due to temperature is a significant concern as global temperatures continue to rise. Studies have shown a positive association between mean temperature and the number of dengue cases, indicating that as global surface temperatures increase, the burden of dengue is likely to intensify (82). Studies done in other countries such as Bangladesh (23) have projected an increase in dengue incidence by the end of the century as a result of temperature increase, and have similarly stated its utility in preparing national authorities to prepare health systems to respond to such changes due to climate change. According to an index projecting vector suitability across different climate scenarios developed by Davis et al. (83) the Philippines will experience a significant increase in dengue vector numbers by 2030, especially in the northern regions and isolated pockets of the country.

Climate change, with its projected increase in global surface temperature and changes in rainfall patterns, will impact the environmental suitability for the growth and survival of dengue viruses and mosquitoes, potentially leading to a higher dengue burden globally, nationally, and locally (17). The utility of projections up to 2,100 lies in the formation of a wide consensus on the use of projection periods, which would facilitate easier comparison of these results (17). The transmission of dengue virus is strongly influenced by hydro-climatic conditions that affect the vector’s life cycle and behavior, indicating that future climate and development scenarios may lead to an increase in the burden of dengue (84). Additionally, the rising infectiousness of dengue has been attributed to factors such as urbanization, population growth, and climate variability and change, all of which create conducive conditions for the proliferation of dengue vectors and viruses (85). These have been known to decrease vector incubation time and increase vector populations (11, 12). Temperature changes under future climate change have the potential to elevate the epidemic intensity of dengue in certain areas, highlighting a new way in which climate change could impact infectious disease transmission (86).

The data projections in the medium-term up to 2030, may serve as a guide in formulating the country’s response for action toward climate change, as the National Climate Change Action Plan is set for review and another iteration in the year 2028 (29). Similarly, the Philippine Development Plan, entitled *AmBisyon Natin 2040* identifies climate change adaptation as one of its key areas of action. These projections may serve as a benchmark and baseline for the progress of the country’s specific initiatives targeted to adapt to climate change (51). Such medium and long-term projections are useful for establishing trends and evaluating the effectiveness of policies and national plans, and in ensuring that strategies are on track to meet their objectives.

### 4.4 On provincial healthcare spending

Socioeconomic factors are relevant in the study of dengue incidence as these are highly linked to behavioral practice and individual susceptibility (87). Our results show an association between healthcare expenditure and disease burden, which was observed in Figure 5, albeit the disparity between low and high spending does not demonstrate a substantial difference in the burden of disease. This may be due to priorities in budget allocation, which favors reactive healthcare over preventive measures. In the Philippine National Health Accounts of 2022, a substantial gap was observed with curative care receiving nearly five times more budget allocation (46%) than preventative care (11.5%) (88). Budget allocations are a reflection of a society’s priorities and are as crucial as increasing budgets in combating disease burdens effectively (89, 90).

Furthermore, disease mitigation efforts seen in initiatives such as the National Program for Dengue Control in the Philippines which centers on the reduction or elimination of vector breeding habitats has struggled to meet its goal of decreasing dengue transmission due to barriers in the implementation and perceived limitations of the strategy (91). A key part of the prevention and control program lies in health communication campaigns which push for community-based interventions and behavior change. However, studies in the Philippines (92, 93) have shown a lack of confidence and awareness in the effectiveness of dengue prevention initiatives at the household level and the belief that decreasing vector habitats should primarily be shouldered by the government.

### 4.5 Limitations

Several limitations must be acknowledged in this study. Firstly, the dengue cases included are limited to those reported by hospitals and health centers, thus excluding cases managed at home, potentially underrepresenting the true burden of dengue, especially in provinces where health centers and hospitals are less accessible. Some provinces exhibited lower attribution rates throughout the year, potentially due to underreporting or data limitations. Moreover, certain provinces were omitted due to insufficient case numbers (less than 300,000). Sensitivity analyses observed that their inclusion would compromise statistical power and potentially introduce bias into the results of the other provinces. Newly formed provinces, such as Davao Occidental and Dinagat Islands, were also excluded due to a lack of historical data, limiting the generalizability of the findings. The role and contribution of non-human reservoirs in dengue cases have also been excluded by this study due to lack of data.

This study did not account for changes in dengue surveillance and reporting between 2010 and 2019. Prior to 2015, dengue surveillance was only done in selected sentinel sites and for clinically-confirmed cases, before the Department of Health mandated nationwide reporting to cover even probable cases (94, 95). Although available to the researcher, dengue incidence data from 2020 to 2022 was deliberately excluded in the analysis to isolate the influence of the COVID-19 pandemic, and the resulting restrictions imposed on dengue risk and reporting, potentially confounding both risk estimation and future burden projection (96, 97).

Additionally, specific socioeconomic variables, including population density, household size, and poverty incidence, were excluded due to concerns of over linearization in risk functions. While their exploration in subsequent studies could provide valuable insights, national-level projections for these factors are currently unavailable. Furthermore, the study focused solely on climatic variables in relation to dengue incidence, neglecting the exploration of other potentially influential climatic factors. Future research may benefit from incorporating a broader range of socioeconomic variables and climatic factors to enhance the understanding of dengue dynamics and their potential utilities in dengue early warning systems.

## 5 Conclusion

The association between dengue fever, vector suitability, and increased temperatures has long been established in existing literature. As climate change proceeds, vector-borne diseases will continue to pose significant public health issues, highlighting the need for efficient climate mitigation strategies to curb future disease burden. This study provides novel insights into the historical excess dengue disease burden attributable to increased temperature as well as country-wide projections to help guide the Philippines’ medium and long-term climate change mitigation plans and interventions. By analyzing a comprehensive 10-year dataset and employing advanced modeling techniques, the study demonstrates that temperature plays a significant role in driving dengue incidence in the Philippines, with 72.2% of cases from 2010 to 2019 attributable to temperature. Notably, the highest attributable fraction occurred between the middle of the warm-dry season and early rainy season, challenging the conventional notion of dengue as primarily a rainy season disease.

Warming temperatures will continue to increase dengue vector suitability, which will affect vulnerable provincial pockets of the country, particularly the conflict-ridden southern, peri-equatorial regions of the country, likely due to increased urbanization outpacing targeted resource allocation for vector control and prevention efforts. If the climate crisis persists, vector suitability stands to significantly increase by year 2,100 compared to the most conservative possible climate scenario. Increasing provincial healthcare spending may also not improve these outcomes significantly if used inefficiently or ineffectively for disease prevention, surveillance, and response.

The novelty of this research lies in its approach to estimating and projecting temperature-related dengue burden, utilizing a comprehensive spatiotemporal dataset, and accomplished both at the national and provincial levels in the Philippines. By incorporating future climate scenarios based on Shared Socioeconomic Pathways (SSPs), the study provides valuable insights for long-term policy planning and public health programming. Our medium-term projections offer guidance for aligning national climate policies, such as the next review of the National Climate Change Action Plan in 2028, and the Philippine Development Plan in 2040, with climate adaptation priorities. These medium and long-term projections may serve as benchmarks for monitoring the effectiveness of targeted initiatives aimed at climate resilience.

Additionally, the short or long-term impact of extreme weather events on vector population and dengue incidence may also provide further insight for preventative vector control and programming. Understanding how the distribution and intensity of diseases like dengue might change, allows for the creation of resilient health systems capable of adapting to a changing climate landscape while also contributing to expanding much-needed evidence emanating from the Global South. These would all provide the potential to guide future scientific efforts aimed at elucidating the impacts of climactic variables on dengue and other vector-borne diseases.

## Data Availability

The datasets presented in this study can be found in online repositories. The names of the repository/repositories and accession number(s) can be found in the article/Supplementary material.
