# Supplementary material for: Projecting temperature-related dengue burden in the Philippines under various socioeconomic pathway scenarios
Source: Front Public Health. 2024 Dec 23;12:1420457. doi: 10.3389/fpubh.2024.1420457 (PMC11701004; doi:10.3389/fpubh.2024.1420457)
Supplement: Supplementary file 1 [file Data_Sheet_1.docx]

***Supplementary Material***

**Figure S1.** Actual (in blue) and extrapolated (red) risk association between temperature and dengue incidence


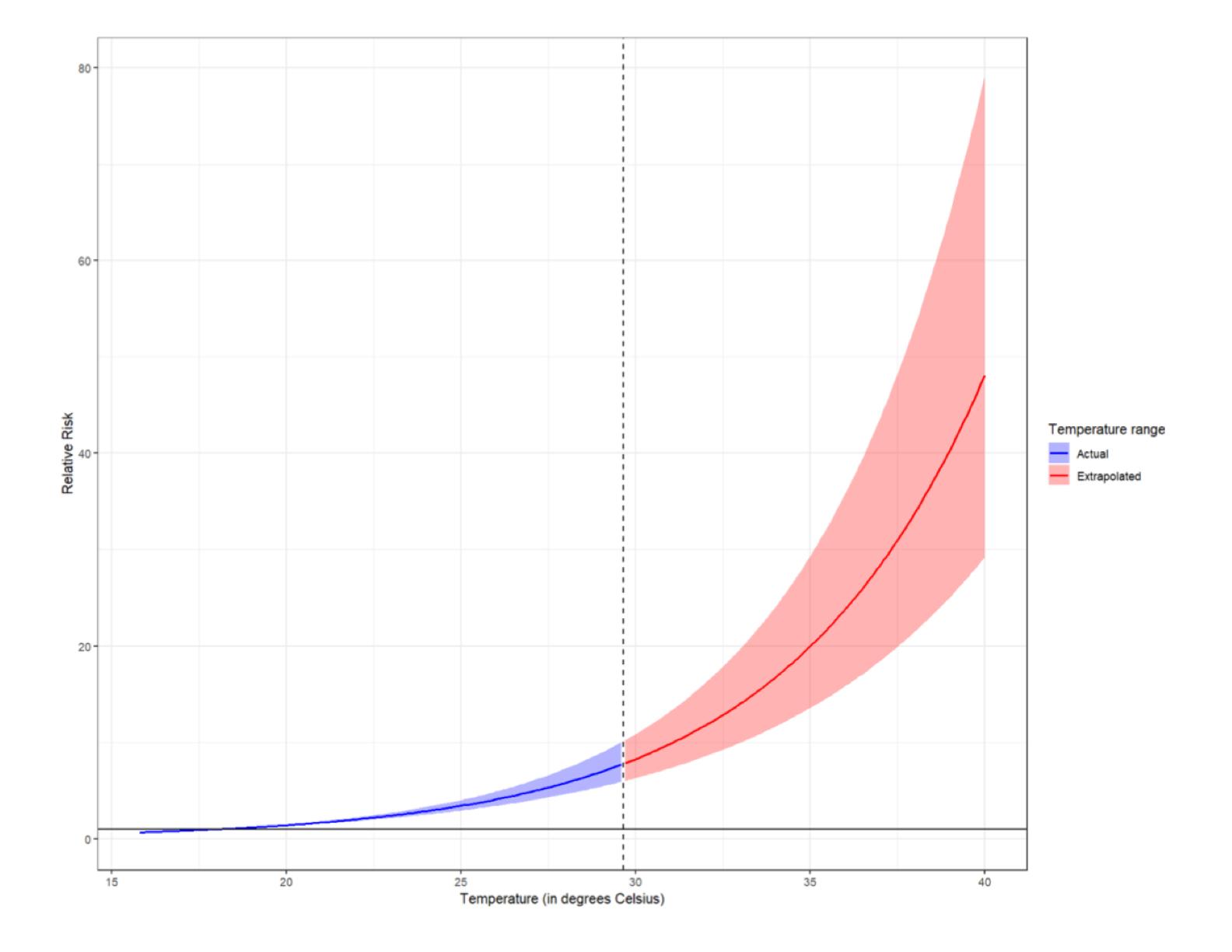


The model used to calculate the dengue incidence projection from our previous paper was recalibrated from a non-linear, U-shape to a linear function to better visualize the future burden of disease (**Fig S1**). The upper and lower limit of the extrapolated projections in red represents the higher (SSP 5-8.5) and more conservative climate scenarios (SSP 1-2.6), respectively. Our results show that relative risk for dengue incidence will exponentially increase under different climate scenarios from an ambient temperature of upwards 29°C. However, as ambient temperatures increase, so will the uncertainty in the projections, as depicted by the enlarging red area, and the central red line may be used as a point of reference for the projected incidence trends.

**Table S1**. Nationwide projection of excess temperature-related dengue cases from 2030 to 2090, by SSP, and corresponding percentage difference from SSP 1-2.6

| **Future Scenario** | **2030** | **2040** | **2050** | **2060** | **2070** | **2080** | **2090** |
| --- | --- | --- | --- | --- | --- | --- | --- |
| SSP1-2.6  Sustainable Development | +7,898 | +9,103 | +10,333 | +11,068 | +11,409 | +11,140 | +10,673 |
| SSP2-4.5 Middle-of-the-road | +8,237  (4.29%) | +10,061  (10.52%) | +11,720  (13.42%) | +13,341  (20.53%) | +14,687  (28.72%) | +15,736  (41.25%) | +15,873  (47.60%) |
| SSP3-7.0  Regional Rivalry | +8,161  (3.34%) | +10,355  (13.75%) | +12,810  (23.97%) | +15,377  (38.68%) | +17,568  (53.98%) | +19,722  (77.03%) | +21,501  (99.93%) |
| SSP5-8.5  Fossil-fuel development | +8,651  (9.53%) | +11,253  (23.62%) | +14,201  (37.43%) | +17,013  (54.2%) | +19,847  (73.95%) | +22,945  (105.96%) | +25,364  (135.85%) |

**Table S1** details further the projected excess in the nationwide burden of temperature-related dengue cases, for every decade from 2030 until 2090, across the different future climate scenarios. Values in parentheses pertain to the percentage difference in the excess number of cases using the most conservative climate scenario as the baseline. Note that the excess burden is computed from the baseline attributable number (AN) of temperature-related dengue cases determined in 2019 (Nationwide AN = 294,165 cases).

**Figure S2**. Nationwide total annual dengue cases


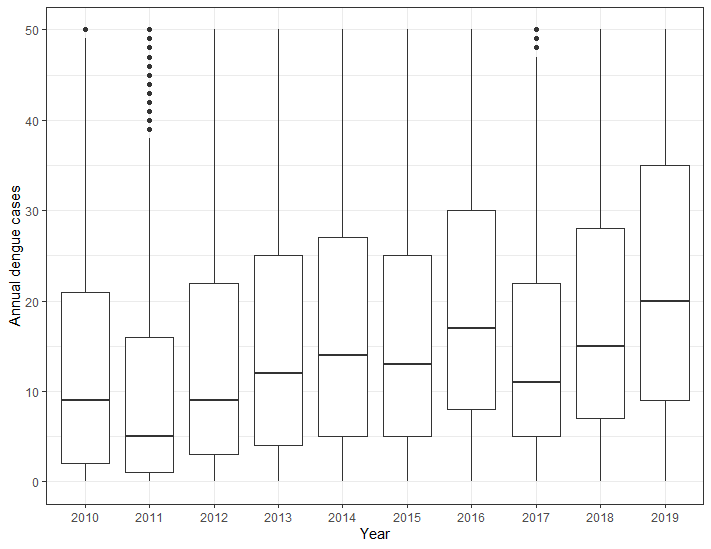


**Figure S2** reveals an annual variability in dengue cases with discernably higher number of cases in 2019 than in earlier periods in 2011 as indicated by the position of the median values depicted by the solid horizontal line in the boxplots.
